# Hearing loss in Peripheral Facial Palsy after decompression surgery

**DOI:** 10.1590/S1808-86942012000300005

**Published:** 2015-10-14

**Authors:** Alexandre Augusto Kroskinsque Palombo, Andre Fernando Shibukawa, Flavia Barros, José Ricardo G. Testa

**Affiliations:** a ENT Physician Graduated from the Otorhinolaryngology and Head and Neck Surgery Program of the Paulista School of Medicine - Federal University of São Paulo (UNIFESP/EPM); Fellowship in Otology - Otorhinolaryngology and Head and Neck surgery program - UNIFESP/EPM.; b Otorhinolaryngologist - Fellowship -Otorhinolaryngology and Head and Neck Surgery Program - UNIFESP/EPM.; c Speech and Hearing Therapist - Otorhinolaryngology and Head and Neck Surgery Department - UNIFESP/EPM.; d Senior Associate Professor of Pediatric Otorhinolaryngology and Otology Program - Otorhinolaryngology and Head and Neck surgery program - UNIFESP/EPM. UNIFESP/EPM.

**Keywords:** bell palsy, decompression, facial paralysis, hearing loss

## Abstract

Facial paralysis can result from a variety of etiologies; the most common is the idiopathic type. Evaluation and treatment are particularly complex. The treatment of acute facial paralysis may require facial nerve decompression surgery. Any structure near the path of the facial nerve is at risk during transmastoid decompression surgery.

**Aim:**

This is a retrospective study, carried out in order to evaluate hearing loss after transmastoid decompression and how idiopathic cases evolved in terms of their degree of paralysis in the last 15 years.

**Materials and Methods:**

We selected the charts from 33 patients submitted to transmastoid facial nerve decompression in the past 15 years and we assessed their hearing loss and facial paralysis.

**Results:**

There was a high percentage (61%) of patients with some degree of hearing loss after the procedure and in all cases there was improvement in the paralysis.

**Discussion:**

The values obtained are similar to those reported in the literature. One possible explanation for this hearing loss is the vibration transmission by drilling near the ossicular chain.

**Conclusion:**

The surgical procedure is not risk free; indications, risks and benefits should be explained to patients through an informed consent form.

## INTRODUCTION

Facial paralysis can be the result of a large variety of etiologies, including infections, neurologic, congenital, neoplastic, traumatic, systemic and iatrogenic^1^. The most common among paralyses is Bell's, or idiopathic, which incidence is estimated to be 2,025 cases per 100,000 inhabitants per year . The peak incidence happens between the second and the fourth decades of life (15 to 45 years of age). There is an equal incidence between genders and facial sides^2^. Facial palsy assessment and treatment are particularly complex, because of the large variation in regeneration potential and the lack of reliable prognostic indicators for spontaneous recovery^3^. The current treatment for facial paralysis is based on a combination of medication, facial physical therapy and surgical intervention in selected cases^4^. The different means of intervention are broken down into acute paralysis (up to 8 weeks), intermediate duration paralysis (8 weeks to 2 years) and chronic paralysis (longer than 2 years), each with their different indications and possible complications. Treatment for the acute facial paralysis may involve facial nerve decompression surgery, primary grafting or repair in cases of resection or transection^4^. Any structure near the facial nerve path is under risk during the nerve decompression surgery via transmastoid approach. May & Klein^5^ reported that hearing impairment was the most frequent complication (air-bone gap, sensorineural hearing loss and lower hearing acuity).

The goal was to evaluate a possible hearing loss after facial nerve decompression via the transmastoid approach and the evolution of the paralysis grade in the idiopathic cases of the last 15 years, operated in our hospital (teaching hospital).

## MATERIALS AND METHODS

We selected the charts from 33 patients in the otology ward of our institution, submitted to transmastoid facial nerve decompression by idiopathic acute facial nerve paralysis in the past 15 years. We compared the degrees of hearing loss (by checking the SRT, sensorineural losses in the low and high frequencies (between 4,000 Hz and 8,000 Hz) and paralysis evolution according to the House-Brackmann scale before and after the procedure. Exclusion criteria: patients previously deaf on the palsy side, incomplete chart or lack of data. This study has been assessed and approved by the Ethics in Research Committee - protocol: CEP 1601/10.

## RESULTS

Among the charts of the patients evaluated, 17 were males (52%) and 16 (48%) were women. Their ages varied between 12 and 66 years, with a mean age of 36.8 years.

### Hearing loss analysis

Sensorineural hearing loss was found in 20 patients (61%), and high frequency hearing loss (above 4 KHz) was present in all the patients. The SRT was the same in 23 patients (70%). Nonetheless, these patients, 10 (31%) had hearing loss in the high frequencies. Of the ten patients with SRT variations, four had a 5dB worsening, five had 10dB worsening and one had a worsening higher than 10dB (Table 1).

**Table 1:** Hearing loss distribution (absolute number and percentage).

Hearing loss	Absolute number	Percentage (%)
5 dB drop in SRT	1	3
10 dB drop in SRT	5	15
SRT drop > 10 dB	4	12
SRT maintained + drop in the high frequency	10	31
No hearing loss	13	39

Total	33	100

### PFP improvement analysis (House-Brackmann scale)

We used the House-Brackmann scale to assess the degree of peripheral facial paralysis. Initially, 29 patients (88%) had grade V PFP; three patients (9%) had PFP grade IV and one patient (3%) had grade VI PFP. After surgery, seven patients (21%), 19 patients (58%) and seven patients (21%) had PFP grades I, II and III, respectively.

## DISCUSSION

This study is in agreement with the epidemiological data of the literature about the age range, involving, above all, the economically active population (mean: 36.8 years), Bell's palsy is a rapidly progressive disorder, evolving during 24 or 48 hours. It is caused by edema, believed to arise from an acute viral infection or the activation of a latent infection^7^. Facial nerve ischemia is then caused by the edema and its compression inside the bony canal^8^. It is different from other causes of facial paralysis, by the lack of trauma and the fast progression of the disease. This fast progression helps differentiate it from facial paralysis secondary to tumors, which evolves slowly along weeks to months. Viral-related facial paralysis is usually self-limited. Depending on nerve edema extension, recovery happens within days, or a few weeks, but it may also take many months in severe cases. Starting steroid treatment early on may reduce the progressive edema, thus reducing even further the nerve damage and accelerating patient recovery.

Studies have shown conflicting results as to the efficacy of the empirical use of antiviral medication. Nonetheless, valaciclovir has been recently associated to facial nerve function improvement in the long run^9^. In patients with partial facial paralysis, recovery is usually satisfactory (grades I or II in the House-Brackmann scale). Recovery can be long and incomplete in patients with grade VI of the scale at paralysis onset. In our study we assessed patients whom most were in grade V. Some authors advocate facial nerve decompression when nerve deterioration is fast and severe^10^. Although the literature supports nerve decompression for severe cases, recovery rates are still good without surgical intervention – often times used in patients with recurrent paralysis^7^. Nonetheless, we found improvements in all operated patients, similar to Yanagihara et al. ^11^findings. In a case-control study they submitted 58 patients with idiopathic paralysis to transmastoid decompression of the facial nerve and found a statistically significant improvement in the House-Brackmann scale 60 days after the procedure.

Thus, surgery is indicated in patients who have more than 90% loss in nerve function when compared to the normal side^7^. In these cases, decompression must be made within two weeks of the complete facial paralysis. This procedure is not risk-free. The more vulnerable structures are the ossicles and the labyrinth, followed by the facial nerve itself^5^. There is very little in the literature about the complications and hearing loss associated with this surgery. In 1983, May & Klein^5^ published that sensorineural hearing loss (in the high frequencies of 4000 to 8000 Hz in the beginning) happened in 51% of the cases, a result which was a little lower than what we found (61%). One possible explanation for this hearing loss, especially in the high frequencies, is the transmission of vibration by reaming bone near the ossicular chain, especially the incus and the bony labyrinth.

## CONCLUSION

This study showed that a large portion of patients submitted to facial nerve decompression to treat Bell 's palsy had some degree of hearing loss after the procedure. Notwithstanding, the proportional rate of loss was low. Thus, this surgical procedure is not risk-free; patients must be clearly informed about its indication, risks and benefits, by means of an informed consent form.
